# Identification of UCP1 and UCP2 as Potential Prognostic Markers in Breast Cancer: A Study Based on Immunohistochemical Analysis and Bioinformatics

**DOI:** 10.3389/fcell.2022.891731

**Published:** 2022-07-07

**Authors:** Xin Yu, Manman Shi, Qi Wu, Wen Wei, Shengrong Sun, Shan Zhu

**Affiliations:** ^1^ Department of Breast and Thyroid Surgery, Renmin Hospital of Wuhan University, Wuhan, China; ^2^ Tongji University Cancer Center, Tenth People’s Hospital of Tongji University, Shanghai, China

**Keywords:** breast cancer, uncoupling protein, tumor immunity, tumor metabolism, thermal tomography

## Abstract

**Background:** Uncoupling protein 1 (UCP1) and UCP2 are associated with tumor metabolism and immunity. However, the prognostic value and molecular mechanisms underlying their action in breast cancer (BC) remain unclear.

**Materials and methods:** In TCGA-BRCA cohort, we investigated the expression characteristics of UCP mRNAs, analyzed their prognostic value by Kaplan-Meier survival analysis, their potential molecular functions by gene set enrichment analysis, and their relationship with immune infiltrating cell types using TIMER and CIBERSORT, along with the assessment of their association with mutational profiles. Kaplan-Meier survival analysis was performed for UCPs in our cohort and their association with BC thermogenesis was assessed by thermal tomography.

**Results:** High expression of UCP1 and UCP2 were positive prognostic markers for BC. UCP1 was associated with the impaired glucose metabolism, while UCP2 with enhanced anti-tumor immunity. High expressions of UCP1 and UCP2 were associated with CDH1 mutations. High UCP1 expression was associated with a high rate of thermogenesis in BC.

**Conclusions:** These results implied a key role of UCP1 and UCP2 in prognosis, metabolism, and immune infiltration in BC. Further investigation of the relevant molecular mechanisms may provide new strategies for BC treatment.

## Introduction

Breast cancer (BC) is currently the most common malignancy and the second leading cause of cancer-related deaths among women worldwide (Sung et al., 2021). According to the recent statistics report, the number of new breast cancer cases has reached 2.3 million in 2020 (Sung et al., 2021). Although the survival outcome of BC patients has shown significant improvement owing to the developments in surgery, chemotherapy, radiotherapy, endocrine therapy, immunotherapy, and other medical technologies, several patients still face the risk of recurrence and death (Emens, 2018; Siegel et al., 2022). Therefore, it remains necessary to develop new biomarkers that contribute to the early detection of BC, thus reducing recurrence and mortality (Weigel and Dowsett, 2010).

Uncoupling protein (UCP) localizes to the inner mitochondrial membrane and decouples oxidative phosphorylation by reducing the mitochondrial membrane potential, thereby directly converting energy, resulting in heat dissipation but without ATP production (proton leakage) (Jacobsson et al., 1985). To date, five UCPs (UCP1–UCP5) have been identified in mammals (Echtay et al., 2018). Among them, UCP1 and UCP2 are associated with multiple malignant tumors, including colon cancer, non-small cell lung cancer, pancreatic cancer, and BC (Ayyasamy et al., 2011a; Li et al., 2013; Pitt, 2015; Giatromanolaki et al., 2017a; Oleksiewicz et al., 2017). Currently, there are different conclusions on the role of UCPs in malignancy. Some studies suggest that UCPs (mainly UCP2) accelerate tumor progression by promoting aerobic glycolysis (Warburg effect) and inhibiting reactive oxygen species (ROS), thereby enhancing the resistance of cancer cells against chemotherapeutic agents (Ayyasamy et al., 2011b; Brandi et al., 2016; Giatromanolaki et al., 2017b; Yu et al., 2020). However, UCP1 reportedly can inhibit tumor progression by initiating autophagy and lipid browning programs (Xiao et al., 2019), along with snail-mediated repression of fructose-bisphosphatase 1 (FBP1) (Zhang et al., 2021). Moreover, evidence suggests that UCP2 enhances anti-tumor immunity in melanoma by reprogramming the immune status of TME (Cheng et al., 2019). Thus, the role of UCPs in BC progression remains unclear.

Given the thermogenic effects of UCPs, these may be associated with cancer thermogenesis. Thermal tomography is an imaging technique based on tumor thermogenesis. It uses infrared radiation to detect the surface temperature of a lesion and further generates a q-r curve from this information to reflect the thermal intensity following the depth of the chromatogram (Shi et al., 2015). Previous experiments confirm that the q-r curve of most BC patients is between 30° and 45°, while that for most benign BC patients is between 15° and 30° (Shi et al., 2015). Thermal tomography has been used for early screening of BC (Sun et al., 2019) and monitoring of drug efficacy in the initial stages (Wu et al., 2017). However, thermal tomography is ineffective in some patients (Wu et al., 2017). Assessing the association of UCP expression with breast malignancy thermogenesis may facilitate the use of thermal tomography in the diagnosis and treatment of BC.

In this study, we performed a bioinformatic analysis for the functions of UCP1 and UCP2 and their prognostic significance for BC patients using data from online databases, validated the prognostic roles of UCP1 and UCP2 in BC in clinical samples collected from these patients, and analyzed the relationship between UCP1 and UCP2 and BC thermogenesis.

## Materials and Methods

### Patients and Specimens

Cohort 1: The gene expression data (RNA-FPKM), information on gene mutation, and clinicopathology of TCGA-BC samples (TCGA-BRCA) were obtained from the UCSC Xena (https://xenabrowser.net/hub/) database. The primary BC (*n* = 1,090) and normal breast tissue (*n* = 113) specimens were included. The illuminaHumanv4.db R package was used to convert the probe IDs to gene symbols. When gene symbol matched more than one probes, the average value was calculated as expression level.

Cohort 2: between February 2015 and October 2017, formalin-fixed paraffin-embedded specimens of 107 patients with BC were collected from Renmin Hospital of Wuhan University. Patients were selected based on their q-r curves at 0°–30° versus 30°–45° in a ratio of 1:1. Patients with at least a 5-year follow-up were included in this retrospective study. All patients were females and provided written informed consent. The study design was approved by the Institutional Ethics Committee of the Renmin Hospital of Wuhan University (No. 2013-081). The endpoint of this follow-up study was recurrence or distant metastasis. Patients’ characteristics are shown in Table 1.

**TABLE 1:** Clinical information of patients in cohort 2. Bold indicates *p*-values less than 0.05.

Characteristics Total (*n* = 107)		UCP1			UCP2		
		Low (*n* = 47)	High (*n* = 60)	*p* value	High (*n* = 67)	Low (*n* = 40)	*p* value
Age				0.40			0.16
<60	91 (85.05%)	42 (39.25%)	49 (45.79%)		60 (56.07%)	31 (28.97%)	
≥60	16 (14.95%)	5 (4.67%)	11 (10.28%)		7 (6.54%)	9 (8.41%)	
Subtype				0.75			0.13
Basal	13 (12.15%)	6 (5.61%)	7 (6.54%)		10 (9.35%)	3 (2.80%)	
Her2	35 (32.71%)	13 (12.15%)	22 (20.56%)		26 (24.30%)	9 (8.41%)	
LumA	23 (21.50%)	10 (9.35%)	13 (12.15%)		12 (11.21%)	11 (10.28%)	
LumB	36 (33.64%)	18 (16.82%)	18 (16.82%)		19 (17.76%)	17 (15.89%)	
ER				0.46			0.07
Negative	51 (47.66%)	20 (18.69%)	31 (28.97%)		37 (34.58%)	14 (13.08%)	
Positive	56 (52.34%)	27 (25.23%)	29 (27.10%)		30 (28.04%)	26 (24.30%)	
PR				0.67			**0.01**
Negative	56 (52.34%)	23 (21.50%)	33 (30.84%)		42 (39.25%)	14 (13.08%)	
Positive	51 (47.66%)	24 (22.43%)	27 (25.23%)		25 (23.36%)	26 (24.30%)	
Stage				0.40			0.35
Ⅰ/Ⅱ	74 (69.16%)	30 (28.04%)	44 (41.12%)		49 (45.79%)	25 (23.36%)	
Ⅲ/Ⅳ	33 (30.84%)	17 (15.89%)	16 (14.95%)		18 (16.82%)	15 (14.02%)	
q-r cruve				**0.02**			**0.04**
0°–30°	49 (45.79%)	28 (26.17%)	21 (19.63%)		25 (23.36%)	24 (22.43%)	
30°–45°	58 (54.21%)	19 (17.76%)	39 (36.45%)		42 (39.25%)	16 (14.95%)	

### Kaplan-Meier Plotter Webtool

The Kaplan-Meier plotter (https://kmplot.com, an online tool for evaluating the association between different gene expression and prognosis of malignant tumors patients based on TCGA, GEO and EGA databases) (Nagy et al., 2021) was used to assess the prognostic value of mRNA expression of UCPs in BC patients. The best cutoff algorithm on the web was used to divide BC patients into high- or low-expression group.

### Functional Exploration

Gene Set Enrichment Analysis (GSEA) was performed by the clusterprofiler R package to analyze gene set enrichment with respect to gene expression based on the Kyoto Encyclopedia of Genes and Genomes (KEGG) and Gene Ontology (GO) annotation results.

### Profiling of Immune Status

Tumor Immune Estimation Resource (TIMER, https://cistrome.shinyapps.io/timer/) was used to calculate the tumor purity and six immune cells, and the Spearman’s correlations between immune cells and gene expression. And the CIBERSORT algorithm was used to calculate the infiltration of 22 immune cells in tumors by cibersort R package.

### Immunohistochemistry

As described in our previous study (Li et al., 2018), IHC staining was performed by two independent pathologists and the staining results were evaluated according to the proportion and intensity of positively stained tumor cells. The intensity of protein expression was scored as 0 (no staining), 1 (weak staining, light brown), 2 (moderate staining, brown), or 3 (strong staining, dark brown). The protein staining score was determined using the following formula: overall score = percentage score × intensity score. Cut-off of the protein staining score of UCP1 and UCP2 were determined by calculating the cut-off values between all possible upper and lower quartiles and selecting the best performing threshold. The antibodies included in this assay were anti-UCP1 (MAB6158, R&D Systems) and anti-UCP2 (ab203244, Abcam).

### Survival Analyses

Survival analyses were performed by the Kaplan-Meier method using survival R package. The univariate Cox regression analysis was performed to identify the association between prognosis of BC patients and clinical factors (including age, molecular subtype and stage) and the expression of UCPs. Significant prognostic factors found in univariate Cox regression analysis (*p* < 0.05) were further evaluated by multivariable Cox regression model using survival R package.

### Characteristics of Thermal Tomography Examination

The thermal tomography system has been described previously (Sun et al., 2019). The thermal tomography system was provided by Prof. Kaiyang Li (Wuhan University, School of Physics and Technology, Department of Electronic Science and Technology). Before the examination, the patient was required to enter a room at a temperature of 25 ± 3°C with no heat source, take the coat off, and rest for 15 min. The patient’s breasts were not touched during rest. During the examination, the patient was required to raise her hands to expose her bilateral breasts. We recorded the anterior, left anterior, and right anterior images of the patient two meters away. Finally, the infrared thermal image was processed and analyzed on the computer processing system. Thermal tomography results of all patients were collected before surgery.

### Statistical Analysis

The means of UCP expression in different groups were compared by one-way ANOVA and student t-test. Chi-square test was perform to evaluate the relationship between the expression of UCPs and the baseline clinical characteristics. Correlation was evaluated by Spearman’s correlation analysis. All statistical analyses were performed using R version 4.0.0 (https://www.r-project.org/). *p* < 0.05 was considered statistically significant.

## Results

### Expression Signatures of UCPs in Breast Cancer

**FIGURE 1:**
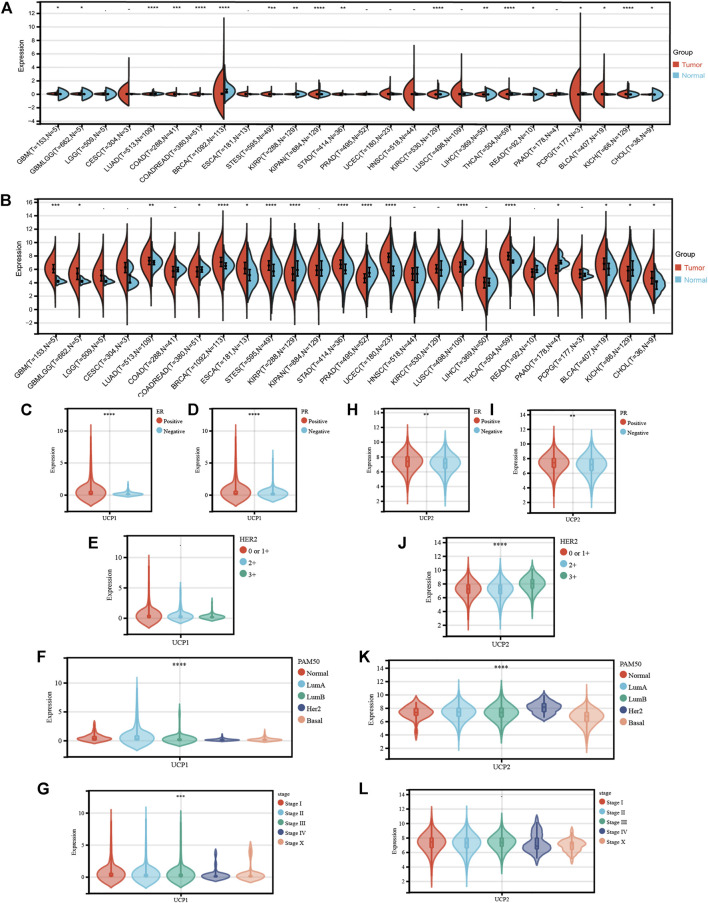
Transcriptional profile of UCP1 and UCP2 in breast cancer. **(A)** UCP1 and **(B)** UCP2 transcriptional expression in 34 cancer specimens as compared to normal tissues from TCGA. **(C–G)** UCP1 transcriptional expression according to different **(C)** ER status, **(D)** PR status, **(E)** HER2 status, **(F)** PAM50 subtype, and **(G)** stage. **(H-L)** UCP2 transcriptional expression for different **(H)** ER status, **(I)** PR status, **(J)** HER2 status, **(K)** PAM50 subtype and **(L)** stage. The statistical difference was compared through the one-way ANOVA and student t-test. *p < 0.05; **p < 0.01; ***p < 0.001; ****p < 0.0001.

We first analyzed the expression of UCP1 and UCP2 between normal and tumor samples from 34 cancer specimens in TCGA (Figures 1A,B). The results showed that UCP1 was significantly up-regulated in 11 tumor types and down-regulated in 7 cancers, including BC. UCP2 was significantly up-regulated in 11 cancers including BC and significantly down-regulated in 6 tumor types.

We then analyzed the expression characteristics of UCP1 (Figures 1C–G) and UCP2 (Figures 1H–L) in BC in cohort 1 (TCGA-BRCA cohort). The result showed that the expression of UCP1 was significantly higher in ER- (Figure 1C) and PR-positive (Figure 1D) patients but not among those with different HER2 statuses (Figure 1E). Among BC patients with different molecular subtypes, UCP1 expression was significantly higher in Normal-like, Luminal A, and Luminal B relative to Her2-enriched and Basal (Figure 1F) groups. UCP1 expression was lower in patients with a higher stage (Figure 1G). UCP2 was highly expressed in ER-, PR-, or HER2-positive patients (Figures 1H–J). UCP2 expression was lowest in Basal and highest in HER2-positive groups among different subtypes (Figure 1K). No significant differences were found in the expression of UCP2 in BC at different stages (Figure 1L).

### Prognostic Value of the UCP Expression in Breast Cancer

**FIGURE 2:**
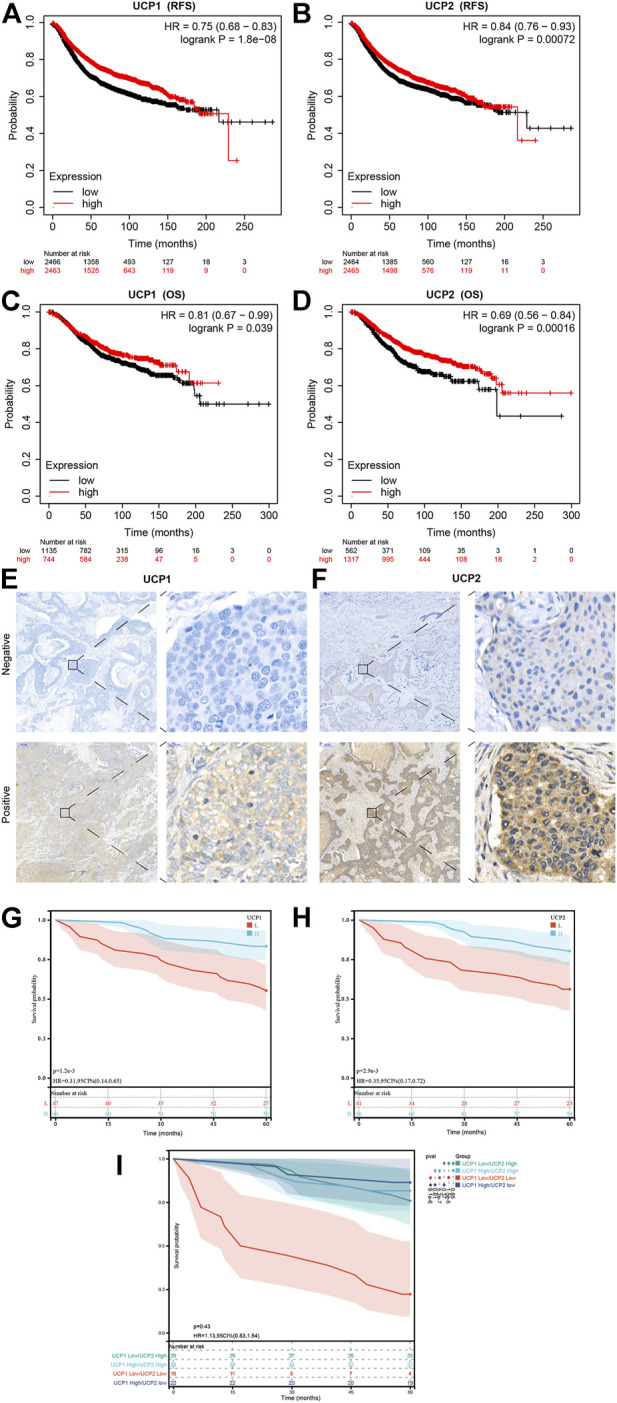
Prognostic value of UCP1 and UCP2 in breast cancer. **(A,B)** RFS curves for **(A)** UCP1 and **(B)** UCP2 in breast cancer using the Kaplan-Meier plotter. **(C,D)** OS curves of **(C)** UCP1 and **(D)** UCP2 in breast cancer using the Kaplan-Meier plotter. **(E,F)** Representative immunofluorescence staining images for **(E)** UCP1 and **(F)** UCP2. **(G,H)** RFS curves for **(G)** UCP1 and **(H)** UCP2 in cohort 2.

The Kaplan-Meier plotter tool was used to examine the association between the expression of UCP1 and UCP2 and the prognoses of BC patients. The results showed that BC patients with high expression of UCP1 or UCP2 had better OS and RFS (Figures 2A–D). We further performed prognostic analysis within different molecular subtypes (Supplementary Figure S1A–T). The results showed that high UCP1 expression was associated with better RFS within different molecular subtypes (Supplementary Figure S1A–E), but only with better OS in the Luminal A subtype (Supplementary Figure S1G). High UCP2 expression was associated with better RFS in Normal-like, Luminal B, Her2-enriched and Basal patients (Supplementary Figure S1K,M–O), and better OS in Normal-like and Basal subtypes (Supplementary Figure S1P,T).

We further evaluated the expression of UCP1 and UCP2 in 107 BC patients by IHC to further verify the association of their expression with the prognosis of BC patients in cohort 2 (Figure 2E,F). The result of IHC showed that UCP1 and UCP2 localized in a diffused manner in the cytoplasm of BC cells. Among the 107 patients, 56.1% (60/107) showed high expression of UCP1, while 62.6% (67/107) showed a high expression of UCP2. Consistently, Kaplan-Meier analysis showed that high expression of either UCP1 or UCP2 was associated with better RFS (Figure 2G,H). The results of Cox regression analysis showed that UCP1 and UCP2 both were independent prognostic factor in BC patient (Table 2). In addition, KM analysis showed that the association with good prognosis was further enhanced when both UCP1 and UCP2 were highly expressed (Figure 2I). These results suggested that UCP1 and UCP2 were markers of positive prognosis in BC.

**TABLE 2:** Cox regression analyses in cohort 2. Bold indicates *p*-values less than 0.05.

Characteristics	Univariate analysis			Multivariate analysis		
	HR	95% CI	p	HR	95% CI	p
Age						
<60 (ref)						
≥60	1.081	0.415–2.816	0.873			
Subtypes			0.811			
Basal (ref)						
Her2	0.953	0.299–3.039	0.935			
LumA	0.712	0.191–2.653	0.613			
LumB	1.190	0.384–3.689	0.764			
Stage						
Ⅰ/Ⅱ(ref)						
Ⅲ/Ⅳ	6.813	3.244–14.306	**<0.001**	8.033	3.728–17.309	**<0.001**
UCP1						
High (ref)						
Low	3.251	1.530–6.908	**0.002**	3.816	1.780–8.182	**0.001**
UCP2						
High (ref)						
Low	2.502	1.233–5.078	**0.011**	4.543	2.042–10.107	**<0.001**

### Functional Analysis of UCPs in Breast Cancer

**FIGURE 3:**
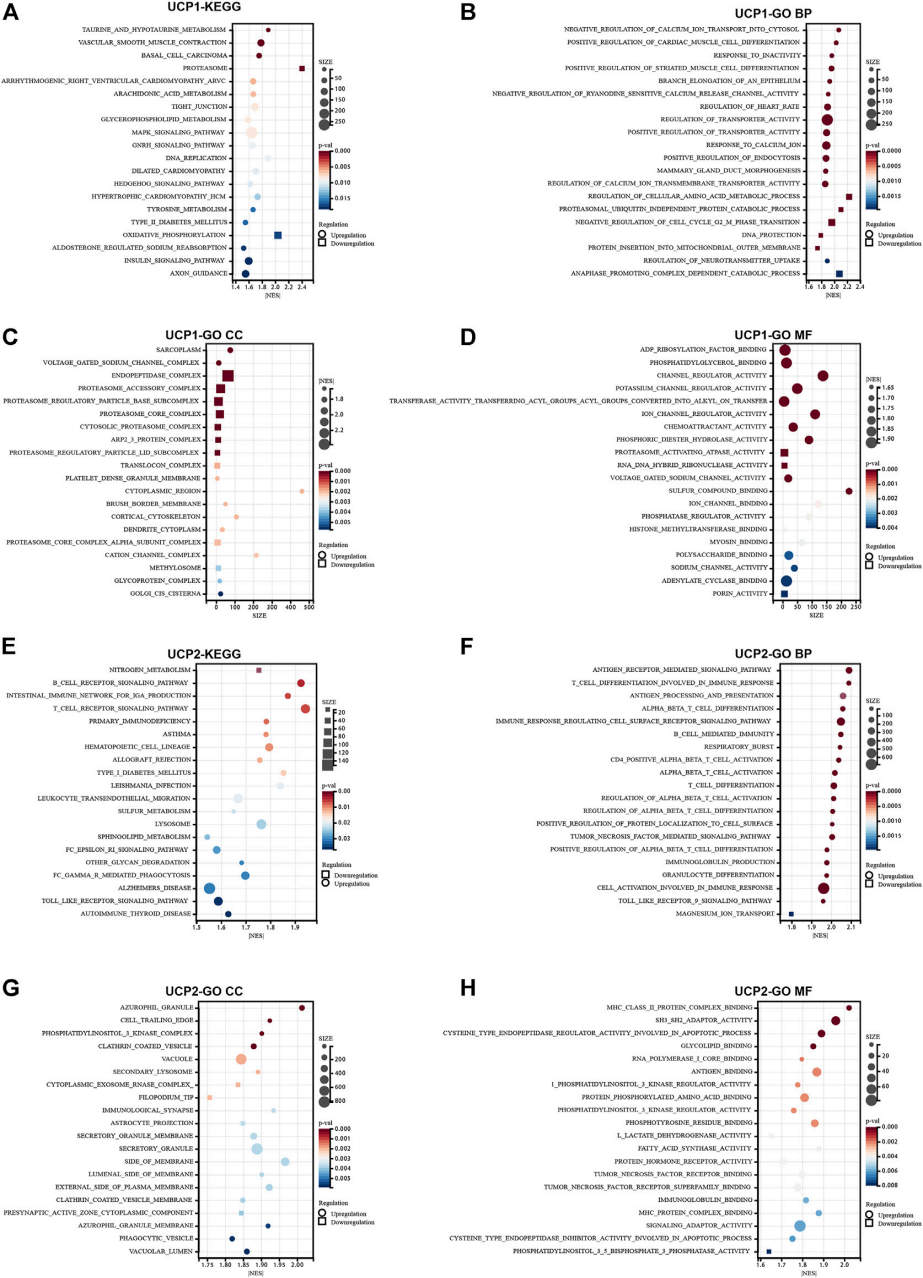
Functional analysis for UCP1 and UCP2 in breast cancer. **(A–D)** Functional analysis for UCP1 by GSEA based on gene sets using **(A)** KEGG, **(B)** GO-BP, **(C)** GO-CC, and **(D)** GO-MF. **(E–H)** Functional analysis for UCP2 by GSEA based on gene sets using **(E)** KEGG, **(F)** GO-BP, **(G)** GO-CC, and **(H)** GO-MF. Only pathways with *p* < 0.05 are presented.

The molecular pathways associated with UCP1 and UCP2 in BC were assessed by GSEA in cohort 1. The KEGG enrichment results revealed the association of UCP1 with multiple tumor metabolic pathways (Figure 3A), including upregulation of taurine and hypotaurine metabolism, arachidonic acid metabolism, glycerophospholipid metabolism, and tyrosine metabolism, and downregulation in oxidative phosphorylation. In addition, UCP1 expression was associated with the downregulation of the DNA replication pathway. GO enrichment results revealed that UCP1 expression was associated with multiple calcium ion regulatory pathways in biological process (BP) terms (Figure 3B). Among cellular components (CC) (Figure 3C), UCP1 was associated with the upregulation of sarcoplasm, the voltage-gated sodium channel complex, and the downregulation of endopeptidase complexes and proteasome-related complexes. In addition, UCP1 was associated with the upregulation of several ion channels among molecular function (MF) terms (Figure 3D).

Pathway analysis revealed a potential link between UCP2 expression and tumor immunity in BC. KEGG enrichment results showed that the UCP2 high expression group showed significantly upregulated immune-related pathways, including B cell receptor signaling, T cell receptor signaling, and leukocyte transendothelial migration (Figure 3E). Consistently, GO enrichment results suggested that UCP2 expression was associated with the upregulation of immune-related pathways, including antigen receptor-mediated signaling, T cell differentiation involved in immune responses, antigen processing and presentation, and alpha-beta T cell differentiation among BPs (Figure 3F); upregulation of azurophil granule, secondary lysosome, immunological synapse among CCs (Figure 3G), and upregulation of MHC class II protein complex binding, tumor necrosis factor receptor binding, and tumor necrosis factor receptor superfamily binding among MFs (Figure 3H).

In addition, we analysed the pathway enrichment associated with both UCP1 and UCP2 altered in BC patients. Consistently, patients with UCP1-High/UCP2-High showed significant alterations in both immune enrichment pathways and metabolic pathways compared to patients with UCP1-Low/UCP2-Low (Supplementary Figure S2). The results of GSEA showed that granulocyte differentiation, production of molecular mediator involved in inflammatory response, CD4 positive alpha beta T cell activation, catecholamine metabolic, secondary alcohol metabolic process and cellular lipid metabolic process were enriched in UCP1-High/UCP2-High groups (Supplementary Figure S2A,B).

We further performed functional analysis of UCP1 and UCP2 in different molecular subtypes (Supplementary Figure S3). The results showed that UCP1 was most associated with proximal substrate tubule bicarbonate reclamation, basal cell carcinoma, Renin angiotensin system, NOD like receptor signalling pathway and long term depression pathway in Normal-like, Luminal A, Luminal B, Her2-enriched and Basal-like subtypes, respectively (Supplementary Figure S3A–E). And UCP2 was most associated with cell cycle, primary immunodeficiency, type II diabetes mellitus, FcεRI signaling pathway and T cell receptor signaling pathway in Normal-like, Luminal A, Luminal B, Her2-enriched and Basal-like subtypes, respectively (Supplementary Figure S3F–J).

### UCPs Associated With Metabolism-Related Genes

**FIGURE 4:**
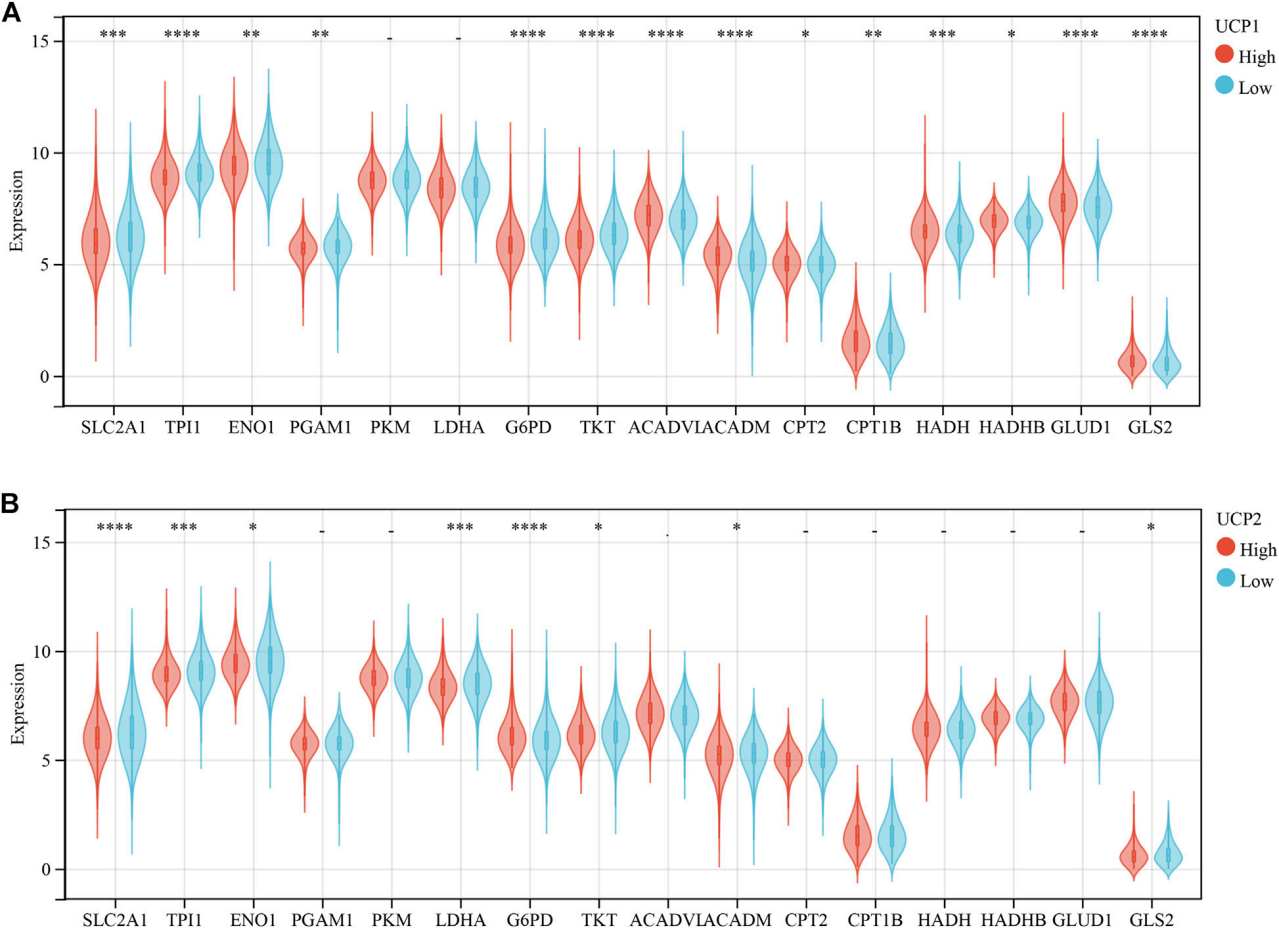
Correlation analysis between UCPs and genes related to metabolism-related gene. **(A)** Correlation analysis between UCP1 and genes related to glycolysis, pentose phosphate pathway, fatty acid oxidation, and glutamine metabolism. **(B)** Correlation analysis between UCP2 and genes related to glycolysis, pentose phosphate pathway, fatty acid oxidation, and glutamine metabolism. The statistical difference was compared through the student t-test. *p < 0.05; **p < 0.01; ***p < 0.001; ****p < 0.0001.

We further examined the association of UCP1 and UCP2 expression with several important metabolic pathway-related genes in cohort 1 (Figure 4). The results showed that UCP1 was negatively associated with several glycolytic or pentose phosphate pathway-associated genes, including solute carrier family 2 member 1 (SLC2A1), triosephosphate isomerase 1 (TPI1), enolase 1 (ENO1), phosphoglycerate mutase 1 (PGAM1), glucose-6-phosphate dehydrogenase (G6PD), and transketolase (TKT). The mRNA levels of several genes involved in fatty acid oxidation (FAO) and glutamine catabolism were significantly and positively correlated with UCP1 expression. UCP2 expression was associated negatively with SLC2A1, TPI1, ENO1, and lactate dehydrogenase A (LDHA) but positively with G6PD. And UCP2 was negatively associated with glutaminase 2 (GLS2).

### Correlation Between UCPs and Immune Status

**FIGURE 5:**
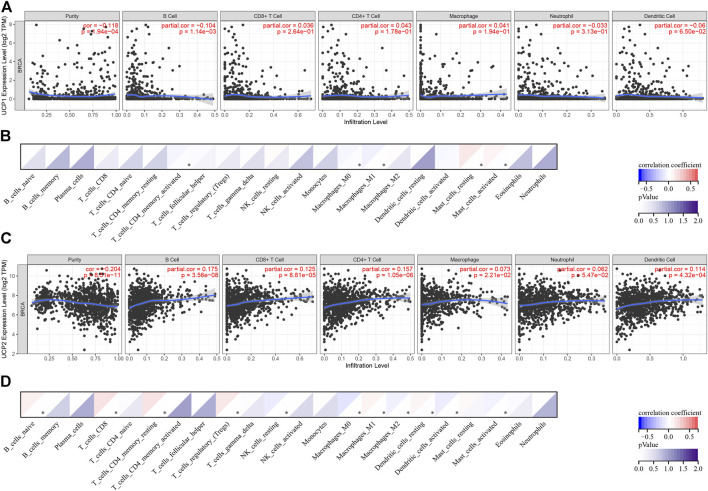
Correlation analysis between UCPs and immune status. **(A,B)** Correlation analysis between UCP1 and immune cell types by **(A)** TIMER and **(B)** CIBERSORT. **(C,D)** Correlation analysis between UCP2 and immune cell types by **(C)** TIMER and **(D)** CIBERSORT. Correlation was evaluated by Spearman’s correlation analysis. *p < 0.05.

The association of UCP1 and UCP2 with the immune status of BC was analysed using TIMER and CIBERSORT in cohort 1. The results of TIMER showed a weak negative correlation between UCP1 expression and B cells (Figure 5A). The results of CIBERSORT showed a negative correlation between UCP1 expression and activated memory CD4^+^ T cells, macrophages M0, and macrophages M1, while a positive correlation was observed with resting or activated mast cells (Figure 5B).

The result of TIMER showed that UCP2 correlated positively with B cells, CD8^+^ T cells, CD4^+^ T cells, macrophages, and dendritic cells (DCs) (Figure 5C). The results of CIBERSORT showed that UCP2 correlated positively with naïve B cells, CD8^+^ T cells, resting memory CD4^+^ T cells, Treg, macrophages M1, and resting DCs, and negatively with resting NK cells, macrophages M0, macrophages M2, activated DCs, and activated mast cells (Figure 5D).

### UCPs and Mutational Landscape

**FIGURE 6:**
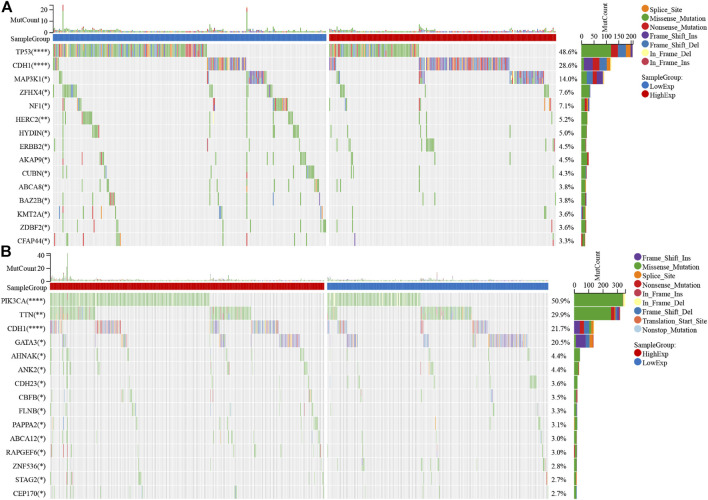
UCPs and mutational landscape. Comparison of the mutational landscape between groups with high- and low- **(A)** UCP1 and **(B)** UCP2 expression. Differences in gene mutation frequency were analyzed by chi-square test. *p < 0.05; **p < 0.01; ***p < 0.001; ****p < 0.0001.

The mutational landscapes for different UCP expression groups were compared in cohort 1. The results showed that both the UCP1-(Figure 6A) and the UCP2-high expression groups (Figure 6B) had a higher frequency of CDH1 mutations. The UCP1 high expression group had a significantly lower frequency of TP53 mutations, while the UCP2 high expression group had a significantly higher frequency of PIK3CA mutations.

### UCPs and Thermogenesis

**FIGURE 7:**
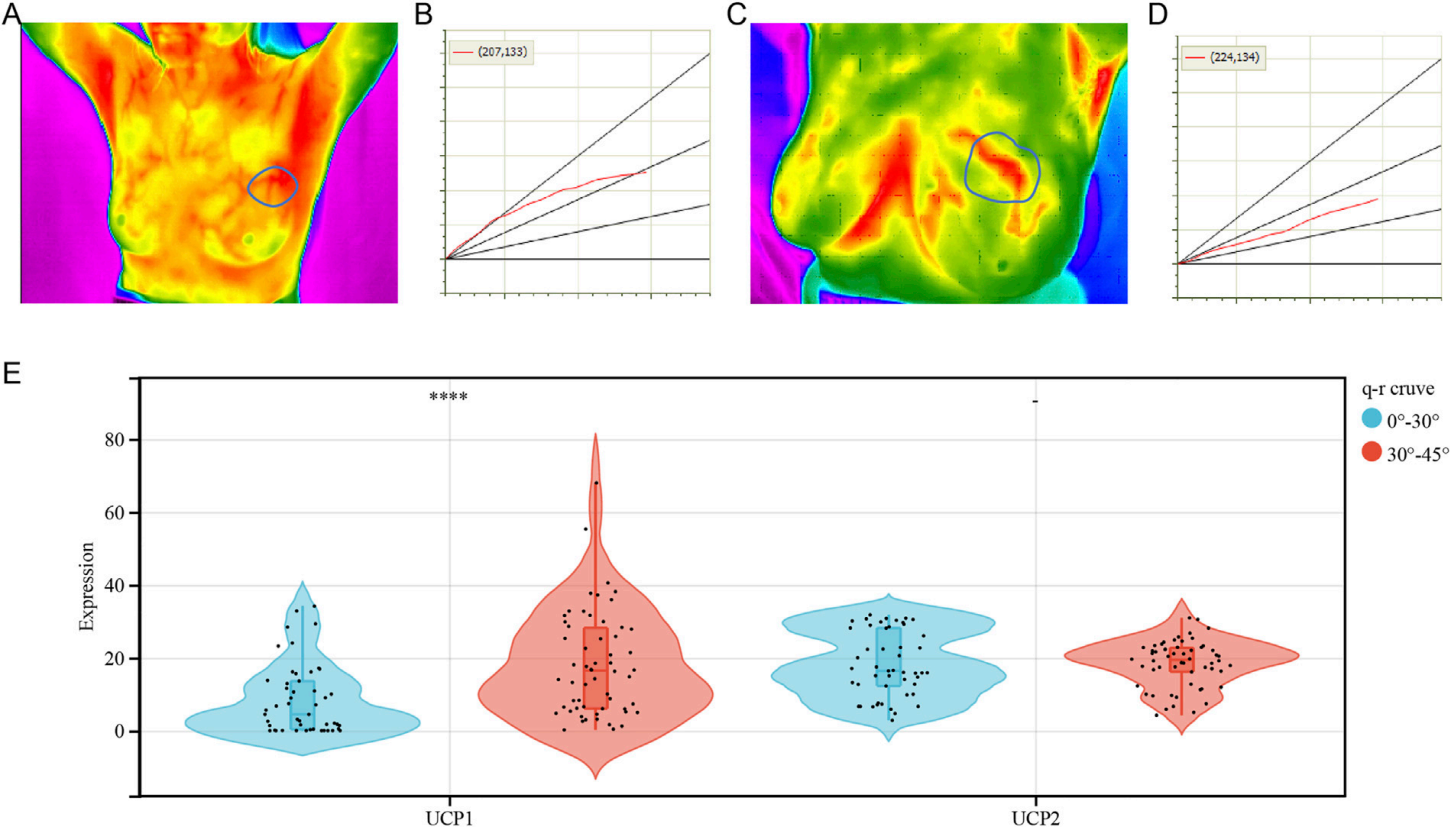
Correlation analysis between UCPs and tumor thermogenesis. **(A,B)** Representative **(A)** heat map and **(B)** q-r curve for breast cancer patients with a high rate of thermogenesis. **(C,D)** Representative **(C)** heat map and **(D)** q-r curve for breast cancer patients with a low rate of thermogenesis. **(E)** UCP1 and UCP2 expression for varying q-r curve status. The statistical difference was compared through the student t-test. *p < 0.05; **p < 0.01; ***p < 0.001; ****p < 0.0001.

To examine the association between UCP1 and UCP2 expression and thermogenesis in BC, we analyzed the correlation between the expressions of UCP1 and UCP2 and thermal tomography results in cohort 2. Most patients with BC had a high rate of thermogenesis (Figure 7A) and q-r curves were at 30°–45° (Figure 7B), while some showed a low rate of thermogenesis with 0°–30° in the q-r curves (Figures 7C,D). The results showed that UCP1 expression was significantly higher in patients with q-r curves at 30°–45°, while UCP2 expression showed no significant differences (Figure 7E). These results suggested that UCP1 expression may be an important factor contributing to the thermogenesis of BC.

## Discussion

UCPs are potential prognostic markers in several types of malignancies, including BC, however, their precise role remains debatable (Ayyasamy et al., 2011a; Li et al., 2013; Pitt, 2015; Giatromanolaki et al., 2017a; Oleksiewicz et al., 2017). Evidence shows that UCP1 has both malignancy-promoting and -suppressing effects. A previous study has shown that UCP1 promotes the catabolism of cancer-associated fibroblasts and cancer-associated adipocytes to provide metabolic energy to cancer cells thereby giving a tumor growth advantage (Wu et al., 2019). Evidence shows that UCP1 inhibits glycolysis in an FBP1-dependent manner in BC (Zhang et al., 2021). UCP1-dependent lipid browning procedure elicits a catabolic state, referred to as “tumor slimming” and inhibits tumor progression [13]. In this study, we demonstrated that BC patients with high UCP1 expression showed enhanced FAO and glutamine metabolism with reduced glucose metabolism, and a better prognosis. Metabolic reprogramming is a hallmark of malignancy (Vaupel et al., 2019). A previous study shows that BC can be classified according to the metabolic profile as glucose-dependent with a poor prognosis or FAO- and glutamine-dependent with a good prognosis (Yu et al., 2021). In addition, evidence showed that UCP1 inhibited glycolysis through an FBP1-dependent pathway (Zhang et al., 2021). Thus, UCP1 expression may serve as an influential factor for the metabolic phenotype of BC, and its potential molecular mechanism of action warrants investigation.

UCP2 also has a paradoxical role in tumor progression. It contributes to tumor progression by promoting metabolism, regulating ROS, preventing tumor apoptosis, and promoting tumor resistance to chemotherapy (Ayyasamy et al., 2011b; Brandi et al., 2016; Giatromanolaki et al., 2017b; Yu et al., 2020). However, evidence also suggests that overexpression of UCP2 does not affect ROS production, but decreases glycolytic activity in cancer cells (Esteves et al., 2014). Moreover, a recent study by Cheng et al. suggests that UCP2 can enhance the anti-tumor responses of CD8^+^ T cells in an interferon regulatory factor 5-dependent manner (Cheng et al., 2019). In the present study, we found that UCP2 was a marker of good prognosis in BC and correlated positively with CD8^+^ T cells as well as M1 macrophages, while negatively with M2 macrophages in BC, which suggested that UCP2 may suppress tumor proliferation by regulating CD8^+^ T cell infiltration and macrophage polarization in BC. Besides, the results of the presented study showed that UCP2 was most strongly associated with a good prognosis in Basal-like breast cancers among the different molecular subtypes. And Functional analysis also showed that UCP2 was more strongly associated with immune activation-related pathways in the Basal-like subtype. As Basal-like breast cancers are more sensitive to immunotherapy (Keenan and Tolaney, 2020), UCP2 may be a target for immunotherapy in Basal-like BCs. Notably, only UCP2 among the UCPs can induce elevated infiltration of T cells in melanoma (Cheng et al., 2019). Similarly, in the present study, the association of UCP1 with the tumor immune microenvironment in BC was minimal in comparison with UCP2. Whether UCP2 induces anti-tumor immunity through molecular functions other than uncoupling oxidative phosphorylation, needs further investigation, as these results may provide new insights into tumor immunity.

Tumor thermography as an imaging technique based on tumor thermogenesis has been widely employed as a screening tool for BC due to the advantages of no-radiation, objective evaluation, and non-invasiveness (Hakim and Awale, 2020; Lozano et al., 2020). In the past, tumor thermogenesis has been mainly attributed to several pro-tumor factors, including tumor metabolism, angiogenesis, and the pro-tumor immune microenvironment (Shi et al., 2015; Gandhi et al., 2021; Lozano et al., 2021). Kawashima et al. reported that UCP1 induced thermogenesis in BC cell lines (Kawashima et al., 2020). In this study, we found that thermogenesis in BC was associated with the high expression of the thermogenic protein, UCP1. The level of UCP1 expression in tumors was significantly lower than that in normal breast tissues and correlated with a good prognosis in BC, which may partly explain the current paucity of evidence available to suggest that intense thermogenesis is a marker of poor prognosis in malignancy. Future research needs to consider the role of UCP1 expression in tumor thermogenesis.

However, this study has some limitations. First, the sample in cohort 2 was selected based on the q-r curve at 0°–30° versus 30°–45° in a 1:1 ratio, which may lead to bias. Second, this was a retrospective study and the findings need to be validated in prospective clinical trials with large patient cohorts. Finally, we only analyzed data from online databases as well as clinical samples, and these conclusions need to be validated in external *in vitro*/*in vivo* experiments and trials.

## Conclusion

In conclusion, high expression of UCP1 and UCP2 were positive prognostic markers for BC. UCP1 was associated with enhanced FAO and glutamine metabolism, while UCP2 with enhanced anti-tumor immunity. In addition, high expression of UCP1 was associated with a high rate of thermogenesis in BC. These findings may provide new perspectives for scientific research on the clinical applications of UCPs.

## Data Availability

The raw data supporting the conclusions of this article will be made available by the authors, without undue reservation.

## Abbreviations

UCP, uncoupling protein; BC, breast cancer; ROS, reactive oxygen species; FBP1, fructose-bisphosphatase 1; GSEA, gene set enrichment analysis; KEGG, kyoto encyclopedia of genes and genomes; GO, gene ontology; IHC, immunohistochemistry; BP, biological process; CC, cellular component; MF, molecular function; SLC2A1, solute carrier family 2 member 1; TPI1, triosephosphate isomerase 1; ENO1, enolase 1; G6PD, glucose-6-phosphate dehydrogenase; TKT, transketolase; LDHA, lactate dehydrogenase A; ACADVL, acyl-CoA dehydrogenase very long chain; ACADM, acyl-CoA dehydrogenase medium chain; GLS2, glutaminase 2; FAO, fatty acid oxidation, DC, dendritic cell.
