# Supplementary figures and images for: Identification of UCP1 and UCP2 as Potential Prognostic Markers in Breast Cancer: A Study Based on Immunohistochemical Analysis and Bioinformatics
Source: Front Cell Dev Biol. 2022 Jul 7;10:891731. doi: 10.3389/fcell.2022.891731 (PMC9300932; doi:10.3389/fcell.2022.891731)

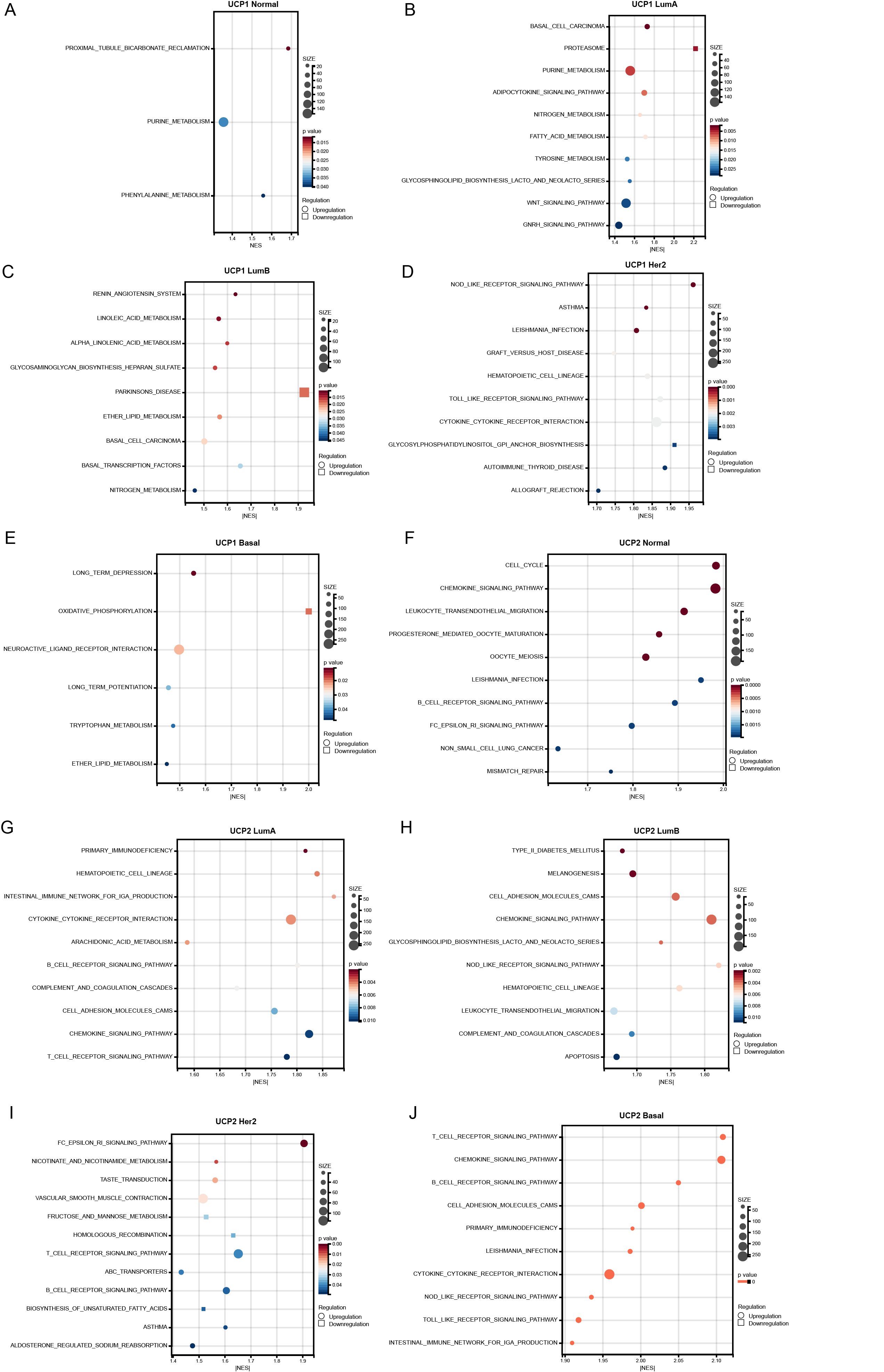

Supplement: Supplementary file 1 [file Image3.TIF]

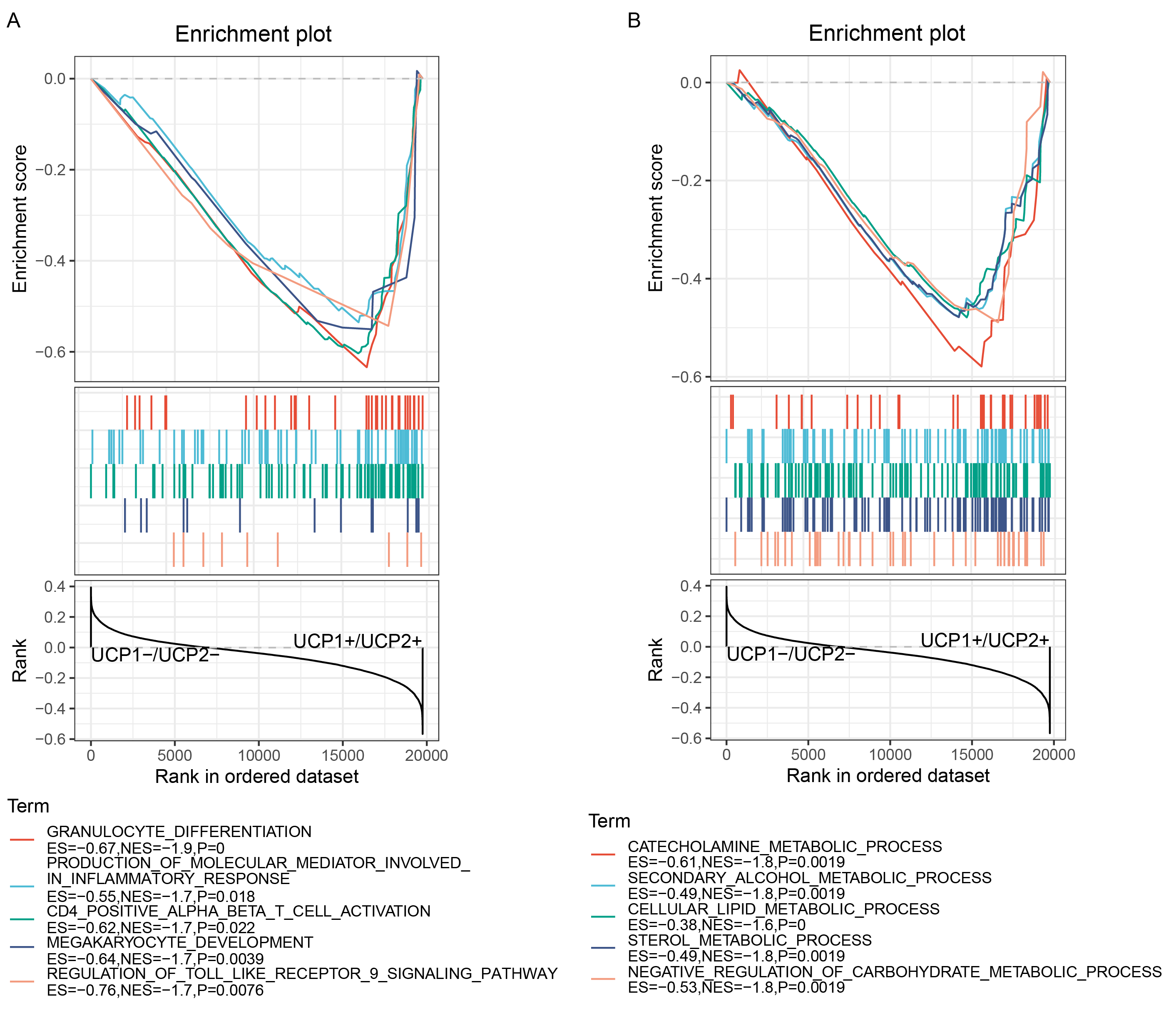

Supplement: Supplementary file 2 [file Image2.TIF]

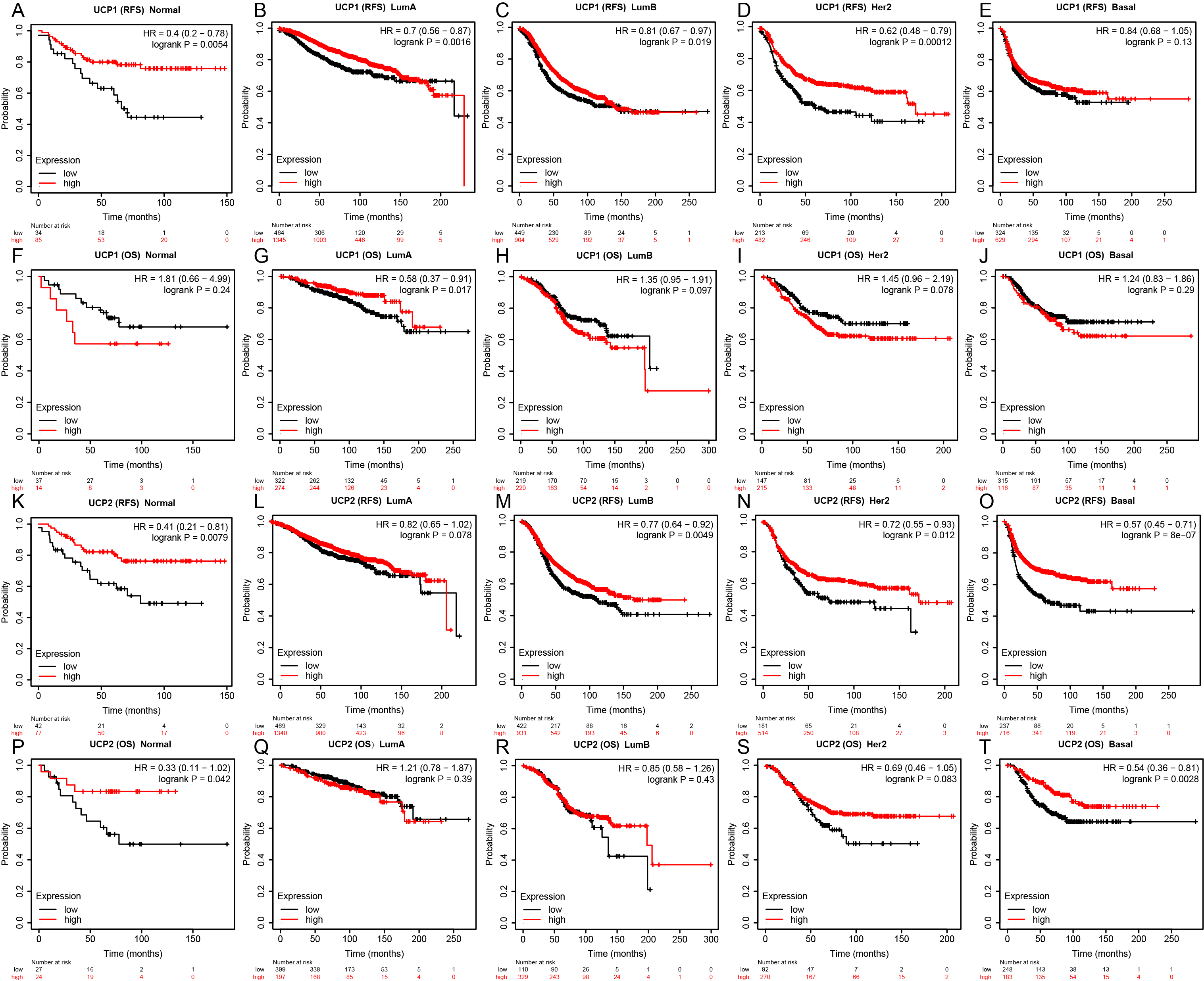

Supplement: Supplementary file 3 [file Image1.TIF]
